# Citizen Scientists as Human Subjects: Ethical Issues

**DOI:** 10.5334/cstp.150

**Published:** 2019-03-08

**Authors:** David B. Resnik

**Affiliations:** * National Institute of Environmental Health Science, US; † National Institutes of Health, US

**Keywords:** citizen science, ethics, human subjects protections, data quality, misconduct

## Abstract

An increasing number of human studies are asking participants to have substantial involvement in research. Citizens in human studies may contribute to various research activities, including study design, recruitment, data interpretation, and data and sample collection. Citizen involvement in research raises novel ethical issues for human studies, because individuals have traditionally occupied the role of researcher or subject, but not both at the same time. The confluence of these two different roles in the same person poses challenges for investigators and oversight committees because legal rules and ethical guidelines focus on protecting the rights and welfare of human subjects and do not address issues that fall outside this domain, such as study design, data quality and integrity, reporting misconduct, authorship, or publication. This article examines some of these issues and makes recommendations for investigators and oversight committees.

## Introduction

The research framework known as citizen science has rapidly increased in prevalence and influence in the last decade (Woolley et al. 2016). Laypeople without formal training in science, i.e., citizens, have helped scientists gather data in disciplines such as zoology, botany, ornithology, astronomy, ecology, meteorology, marine biology, microbiology, and ethology, to name but a few (Silvertown 2009; Dickinson et al. 2010; Resnik et al. 2015a; Garbarino and Mason 2016). For example, in 2016, more than 73,000 laypeople from the United States, Canada, the Caribbean, and Latin America participated in the National Audubon Society’s 117^th^ annual Christmas Bird, providing data on 65 million birds (LeBaron 2017). The bird count helps scientists to inventory bird populations and species. This information can help scientists learn more about bird evolution, adaptation, and migration.

Citizens have also initiated their own clinical research projects (Bottles 2011). For example, in 1994, Sharon Terry formed PXE international, a non-profit organization that advocates for and supports research on a rare genetic disease known as pseudoxanthoma elasticum (PXE), when her two children were diagnosed with the illness. She raised money for PXE research, recruiting families to participate in studies, and patented a genetic test for the disease (Genetic Alliance 2017). As another example, an organization started by chronic pain sufferers Alexandra Carmichael and Daniel Reda, known as Cure Together, invites patients to share their perceptions of the effectiveness of medical treatments for over 500 diseases. Cure Together shares these data with its members and clinical researchers (Cure Together 2018).

Citizen science raises novel ethical and policy issues for research with human subjects, because individuals have traditionally occupied the role of researcher or subject, but not both at the same time. The confluence of these two different roles in the same person poses challenges for investigators and oversight committees because legal rules and ethical guidelines focus on protecting the rights and welfare of human subjects and do not address issues that fall outside this domain (Rothstein et al. 2015). This article examines some of these issues and make some recommendations for investigators and oversight committees.

## Citizen Involvement in Scientific Research

To guide the discussion of ethical issues, I distinguish between the different ways that citizens can be involved in scientific research. When citizens assume a passive role in research, i.e., when they are only research subjects, the main ethical issues concerning their participation involve protection of their rights and welfare. When they assume a more active role, however, the ethical issues concerning their participation also include those that occur in the conduct of science, i.e., research integrity issues. These include: Designing research; preventing, reporting, and investigating misconduct; promoting objectivity in research (conflict of interest); collecting and sharing data and samples; assigning authorship; reviewing and publishing research; mentoring students and trainees; and acting in a socially responsible manner (Shamoo and Resnik 2015). Committees that review and oversee research typically focus on protecting the rights and welfare of participants and usually do not deal with research integrity issues related to participation in research. Thus, citizen involvement in scientific research raises novel issues concerning the review and oversight of research involving human subjects.

Many different types of research projects involving human subjects could be viewed as citizen science. Those with the most active roles for citizens include **citizen-initiated projects** in which laypeople choose the research problem, define aims and objectives, obtain funding, assemble a research team, design the study, collect data, and recruit patients (Bottles 2011, Howes 2016). A somewhat less active role for citizens occurs in **community-based participatory research**, in which investigators select the research problem and obtain funding, but work closely with community members in numerous aspects of the project, including refinement of aims and objectives, research design, survey development, recruitment, informed consent, data collection, and data interpretation (O’Fallon and Dearry 2002; Horowitz et al. 2009; Resnik 2015). A more passive role includes **citizen-assisted projects** in which laypeople help with data or sample collection or recruitment, but have little role in study design, data interpretation, or research tasks that involve substantive intellectual input (Morgan et al. 2014; Hoffman et al. 2015).

## Human Subjects Protections

Various laws and ethical guidelines govern research involving human subjects. These includes regulations, such as the Common Rule (Department of Homeland Security et al. 2017), which applies to federally funded research in the U.S., as well as international guidelines, such as the Nuremberg Code (1949) and the Declaration of Helsinki (World Medical Association 2013). These ethical and legal standards embody the following principles for research with human subjects (Emanuel et al. 2000; Shamoo and Resnik 2015):

1. Scientific rigor, i.e., the research should be well-designed to address important scientific or social questions.
2. Social value, i.e., the research should be expected to yield results that can benefit society.
3. Risk minimization, i.e., the research should minimize risks to human subjects and others.
4. Reasonableness of risks, i.e., the risks of the research should be justified in terms of the potential value of the research to the subjects and society.
5. Informed consent, i.e., informed consent from the subjects or their legal representatives should be sought and appropriately documented.
6. Confidentiality/privacy, i.e., the confidentiality and privacy of the subjects should be protected.
7. Equitable selection of subjects, i.e., the selection of research subjects for study participation should be based on sound scientific and ethical reasons.
8. Data and safety monitoring, i.e., where appropriate, study data should be monitored to protect human subjects from harm or to promote their welfare.
9. Protection of vulnerable subjects, i.e., the research should include additional protections for subjects who may be vulnerable to coercion, exploitation, or harm.
10. Independent review, i.e., the research should be reviewed and overseen by an independent committee, such as an institutional review board (IRB) or research ethics board (REB).

This article will not address each of these principles but will focus on those that are implicated in citizen science involving human subjects.

## Study Design

Good research design is an important principle of ethical research with human subjects, since poorly designed studies may not yield valuable results that justify exposing participants to risks (Emanuel et al. 2000). If a study is poorly designed, the data it generates may be disorganized, uncontrolled, and uninterpretable. Studies should have clearly defined aims and objectives that address important scientific or social questions and should use appropriate methods, tests, or procedures to achieve their aims and objectives, minimize bias, and maximize reproducibility (Shamoo and Resnik 2015). Studies should also include clear descriptions of variables, outcomes, target populations, study personnel, recruitment and consent plans, timetables, sample sizes, statistical analysis plans, and procedures for reporting adverse events and relevant results, non-compliance, and unanticipated problems.

Citizen-initiated studies may have significant problems with study design because laypeople may lack a basic understanding of scientific methods, which can impact all aspects of research including study design, recruitment, data collection, data analysis, and data interpretation. Laypeople also may lack the knowledge, expertise, or experience needed to develop a rigorous research protocol even when they have some familiarity with scientific methods (Bottles 2011; Stone 2013). One way to overcome such problems is for citizens who initiate research to collaborate with professional scientists, to help them learn about scientific methodology and rigorous study designs and research protocols. Sharon Terry, for example, worked with professional scientists to help her develop a genetic test for PXE (Genetic Alliance 2017). Ideally, citizen-initiated studies should undergo some form of scientific review prior to IRB or REB review (discussed below).

## Quality of Data and Sample Collection

Another novel issue relates to the quality of data and sample collection, which is an important concern from the perspective of research integrity as well as human subjects ethics, since problems with the execution of a study can compromise its potential benefits and ethical rationale (Emanuel et al. 2000). Citizens who are recording data or collecting samples need to have appropriate education or training, and sufficient expertise among study staff or supervisors, to ensure that they perform such tasks correctly (Resnik et al. 2015b; Kosmala et al. 2016). In some cases, the education or training need only be minimal. For example, researchers who ask citizens to collect urine and saliva samples could provide them with home collection kits, user-friendly instructions, and a phone number to call with questions. In other cases, more extensive training may be required. For example, citizens who keep diaries concerning their use of consumer goods containing potentially toxic chemicals (such as pesticides and cleaning products) and submit their entries to investigators by means of a cell phone or personal computer may require considerable instruction on how to enter data in diaries and submit them properly (Rothstein, Wilbanks, and Brothers 2015).

When IRBs review problems and concerns related to data or sample collection, they usually focus on matters related to education and training of research staff, not on training of human subjects. But when subjects are also members of the research team, IRBs may need to consider whether the subjects require more training and how the investigator should respond if a subject fails to comply with the protocol by collecting data or samples improperly. Under normal circumstances, a non-compliant staff member could be disciplined as part of a corrective action plan for the study. However, acting against a non-compliant human subject could cause them psychological distress, such as shame or embarrassment. Would it be appropriate for an investigator to withdraw a subject from a study for improper data or sample collection? This is not an easy question to answer because it involves a conflict between the participant’s dual roles (i.e., human subject and member of the research team). Perhaps the best way of dealing with this issue would be to inform subjects during the consent process that they may be withdrawn from the study if they fail to comply with data/sample collection protocols and standard operating procedures. Letting subjects know up front about their data/sample collection responsibilities may help to minimize this type of distress.

## Misconduct

U.S. research regulations prohibit investigators from committing misconduct in federally funded studies. Misconduct is defined as “fabrication, falsification, or plagiarism in proposing, performing, or reviewing research, or in reporting research results…. Research misconduct does not include honest error or differences of opinion (Office of Science and Technology Policy 2000: 76263).” However, many research institutions have definitions of misconduct that include misbehaviors other than fabrication, falsification, and plagiarism, which may need to be accounted for in citizen science. Some of these other misbehaviors include serious deviations from accepted practices, data manipulation, misuse of confidential information, and unethical authorship (Resnik et al. 2015c). Citizen scientists and the researchers who work with them should be aware of applicable misconduct definitions.

Scientists who are found to have committed misconduct may face disciplinary action from their institution, such as a letter of reprimand, increased supervision of research, or termination of employment, as well as discipline from the federal agency, such as a temporary or permanent ban on receipt of federal funding and a requirement to retract affected publications (Shamoo and Resnik 2015). Misconduct investigations are legal proceedings that can cost considerable time, effort, and money for the institution and the involved parties (e.g., accused, accuser, witnesses, and investigators). Federal regulations include provisions for protecting the rights of the accused and accusers (i.e., whistleblowers) and ensuring due legal process (Shamoo and Resnik 2015).

When an investigator suspects that a research staff member who is not also a human subject has fabricated or falsified data, the pathway to resolution is fairly clear: The investigator should report the misconduct to their institutional officials in charge of handling research integrity issues (such as research ethics or compliance officers) as well as the human subjects committee (e.g., the IRB). Both groups would handle the investigation and coordinate with each other. The research integrity officials could appoint a committee to conduct an inquiry into the matter and then appoint another committee to investigate it, if the inquiry committee determines there is sufficient evidence to proceed to an investigation (Shamoo and Resnik 2015). The IRB could treat the matter as an unanticipated problem involving serious non-compliance and could act to protect human subjects and the integrity of the research. The IRB could suspend the study temporarily while the allegation is under investigation, for example, and require the investigator to inform subjects about the problem (Shamoo and Resnik 2015).^1^

Matters could become much more complicated if the individual accused of misconduct is also a human subject, since an investigation could threaten their rights or welfare. Also, false accusations of misconduct can be just as stressful for the accused parties as confirmed allegations. The human subjects committee would need to coordinate with research integrity officials to ensure that human subjects are treated appropriately and not falsely accused. Because the human subjects are not likely to be employees of the institution or interested in receiving federal research funding, disciplinary actions taken by the institution or the federal agency may have little impact on them. Accused subjects might decide to withdraw from the study (if they haven’t been withdrawn already), and they might refuse to cooperate with investigating officials to avoid further psychological distress or legal fees. Ideally, the institution should offer to compensate human subjects from harms related to false accusations. For example, they could offer to pay expenses related to legal fees or psychological counseling. Because individual citizens or even groups of citizens may be motivated to manipulate data to achieve outcomes that promote their interests (such as showing that a chemical in the environment is unsafe or that medication is effective), misconduct by citizens is a possibility that should not be dismissed lightly (Resnik et al. 2015a). Currently there is no documented evidence of any case of research misconduct involving citizen scientists in human studies. Because a real case would raise novel and vexing issues, investigators and oversight committees should plan for this possibility in advance (and map out strategies for responding to accusations if they arise).

## Access to Data and Results

One of the basic tenets of good scientific practice is that investigators and staff members should have access to the data collected by the research team. Data access is necessary for performing various research activities, such as auditing, editing, cleaning, analyzing, and interpreting data, and promoting accountability and trust (Shamoo and Resnik 2015). However, granting human subjects who collect data for a study access to data (besides their own) threatens the confidentiality and privacy of other subjects who are in the study. Researchers could deal with the conflict by allowing human subjects to have access only to their own data or allowing them to have access to other subjects’ de-identified data (i.e., data with personal identifiers removed). The former option is not consistent with treating the human subjects as full members of the research team, and the latter may not adequately protect confidentiality and privacy, because subjects may be able to identify their fellow participants in the de-identified data. For example, if a subject learns that a data set belongs to a white female who is 6’1” and weighs 135 pounds, he or she might be able to identify the subject if they have both visited the same clinic or research center or they both live in a small town, because very few women would fit this description. Investigators and oversight committees will need to resolve these issues when citizens have substantial involvement in data collection in human studies.

Sharing individualized results with participants is a significant ethical issue in research with human subjects (Secretary’s Advisory Committee on Human Research Protections 2017). There is broad consensus that participants should receive clinically useful results from well-validated tests because these results can be helpful in diagnosing, preventing, or treating diseases (Beskow and Burke 2010; President’s Commission for the Study of Bioethical Issues 2013).^2^ For example, participants should receive results related to blood pressure, heart rate, blood sugar, cholesterol levels, and other clinically useful pieces of information.

However, researchers and ethicists disagree about whether participants should receive results that are *not* clinically useful. One of the main arguments against sharing such results with participants is that this may produce more harm than good. For example, a research participant who receives a test result indicating that he has a gene that increases his risk by 15% of developing a rare neurological disease which is currently not treatable or preventable may not understand what the result means or how to act upon it. Receiving this result could cause needless stress and worry without promoting health or well-being. A key argument for returning all well-validated results, even those which are not clinically useful, is that information can enhance autonomous decision-making (Shalowitz and Miller 2005). A second argument is that people have some ownership over this information because it has come from their bodies, tissues, or cells. A third argument is that most people want to receive their individualized tests results (Shalowitz and Miller 2005).

Citizen involvement in biological sample collection may provide an additional reason for sharing all well-validated results with study participants. A person who collects a biological sample is more likely to want to know the test result than someone who passively undergoes a procedure, because he or she has put more time and effort into the research. Also, citizens who collect samples may feel more ownership over the sample than someone who passively undergoes a procedure, because they have invested labor in collecting the sample. Investigators should therefore reflect upon these ethical considerations when deciding whether to share individualized results with citizens who collect biological samples for a study.

## Authorship and Acknowledgement

Citizens who contribute to scientific research may be named as authors or recognized in the acknowledgments section of a paper (Resnik et al. 2015a). Authorship credit or an acknowledgment (whichever is appropriate) is important for 1) expressing gratitude for an individual’s contribution to a project; 2) allocating credit fairly (give credit where credit is due); 3) ensuring accountability for the research (i.e., authorship entails study-related responsibilities); and 4) building trust among citizens and scientists (Resnik et al. 2015b). Most biomedical research journals follow authorship guidelines developed by the International Committee of Medical Journal Editors (ICMJE) or have similar requirements. According to the ICMJE, authorship should be based on fulfillment of each of the following criteria:

Substantial contributions to the conception or design of the work; or the acquisition, analysis, or interpretation of data for the work; AND Drafting the work or revising it critically for important intellectual content; AND Final approval of the version to be published; AND Agreement to be accountable for all aspects of the work in ensuring that questions related to the accuracy or integrity of any part of the work are appropriately investigated and resolved (ICMJE 2017).

Individuals who do not qualify for authorship but make substantial contributions may be named in the acknowledgments section of a paper (ICMJE 2017). The primary question to ask when considering whether citizens should receive authorship credit is whether their contributions have been substantial. Contributions from individual citizens usually are not large enough to meet the threshold for substantiality, because a project may involve dozens or hundreds of citizens. For example, if an environmental health study includes samples and data gathered by 200 citizen scientists, no contribution from a single person would be substantial even though the contribution from the entire group would be. In a case like this, investigators could give authorship credit to the entire group or mention the group in the acknowledgments (Resnik et al. 2015a). In the rare case that a single human subject makes a substantial contribution, he or she could be granted authorship or recognized in the acknowledgments section.

Assignment of authorship in scientific research is far removed from the issues usually focused on by human subjects committees. However, assignment of authorship could become a human subjects issue if a citizen scientist complained that he or she had not received appropriate recognition for his or her contributions to a project. In this situation the human subjects committee could require the investigator to give the citizen proper credit as a way of demonstrating gratitude and respect for the citizen, which could include authorship or an acknowledgment, depending on the case. If a paper already had been published, the investigator could submit a correction to the journal. To avoid misunderstanding, investigators should discuss credit assignment issues with citizen scientists during the informed consent process and at the beginning of the research project.

Researchers sometimes publish photographs of human subjects in scientific journals. For example, a researcher might publish a photo of a skin infection to show what it looks like. Researchers should obtain consent to use such photographs in publications and should remove or obscure identifying features (such as facial characteristics) to protect individual privacy.

## Publication

Publication is important for ensuring that research with human subjects yields socially valuable results, and is required by funding organizations (Shamoo and Resnik 2015a). Ethical issues may arise, however, if citizen participants object to publishing some of the results of research. This type of issue arises more commonly in community-based participatory research than in other types of citizen science involving human subjects (Resnik and Kennedy 2010). For example, suppose that a study that examines access to care and health outcomes in a rural Appalachian county includes a community advisory board that has helped the investigators with survey design, recruitment, and local outreach. The investigators discover that the community has unusually high rates of sexually transmitted diseases and drug/alcohol abuse and report these findings to the board. The board requests that the investigators refrain from publishing these findings, because they are concerned that sharing this information with the public could lead to discrimination and bias against the community. What should the investigators do? On the one hand, they have an obligation to publish their results to enhance human knowledge and thereby benefit society and possibly the local community. On the other hand, they have an obligation to avoid harming the community and to respect the wishes of the board members (Resnik and Kennedy 2010).

Dilemmas like this one are not easy to address, because they involve a conflict between the obligation to protect the community and the obligation to benefit society (Sharp and Foster 2000; Wallwork 2008; Resnik and Kennedy 2010; Terry et al. 2012). Perhaps the best way for investigators to deal with such situations is to involve community representatives in decisions relating to the dissemination of knowledge and information throughout the research process. Publication of results could be addressed both prior to launching and throughout a study to allow investigators and community representatives to develop an understanding of the benefits and risks of publication and how to address community concerns. In some cases, it may be possible to minimize risks by withholding the name and precise location of the community from the publication to protect its anonymity. Also, community members often recognize that the benefits of publication outweigh the risks for the community. For example, publicizing a community’s incidence of sexually transmitted diseases may help to leverage public or private funds to help the community address this problem (Resnik and Kennedy 2010).

## Review and Oversight

Some citizen-initiated projects have been conducted without oversight by an IRB or other human subjects protection committee (Stone 2013). This omission can occur in the U.S. due to a coverage gap in federal research regulations, which cover federally funded human studies as well as privately funded studies that collect data for products regulated by the Food and Drug Administration (FDA) or Environmental Protection Agency (EPA) (Shamoo and Resnik 2015). Or they can result from innocent ignorance on the part of the citizen researcher. Most academic institutions apply federal research regulations (such as the Common Rule) to all human studies on campus, regardless of the source of funding (Klitzman 2015). Thus, if a study is conducted outside of an academic institution, is not federally funded, and is not related to an FDA or EPA-regulated product, it need not undergo IRB review.

Independent review and oversight is a key principle of ethical research with human subjects because investigators have an inherent conflict of interest that may prevent them from recognizing or appreciating ethical concerns in their own research. Investigators also may lack the knowledge and experience needed to address ethical or legal issues related to their studies (Emanuel et al. 2000). Most scientific journals also require authors who submit articles describing human studies to provide information concerning ethical oversight of the research or explain why none was needed (Shamoo and Resnik 2015).^3^

Citizens who initiate their own research involving human subjects should ensure that their projects have independent review and oversight. Because forming their own IRB to review research would be prohibitively expensive, they might decide to submit their proposed projects to a private IRB for review. However, this option could be expensive, because these companies typically charge thousands of dollars for review and oversight of research (Stone 2013). A less expensive option would be to collaborate with an investigator at an academic institution who could submit the project to his or her IRB free of charge. This option also has the advantage that the investigator’s institution might be able to provide scientific review for the study (see discussion of design issues above).

## Conclusion

The confluence of divergent roles—citizen and scientist—creates novel ethical issues when laypeople conduct research involving human subjects. These issues go beyond the human protection concerns (e.g., risk/benefit, consent, confidentiality) typically addressed by oversight committees and extend to topics that fall under the rubric of research integrity, such as study design, data and sample collection, reporting misconduct, data access, authorship, and publication. Investigators should be mindful of these issues when they develop research protocols that are likely to substantially involve citizens in the research process, and IRBs should pay attention to them when they review protocols. Since citizen involvement in research on human subjects is a relatively new trend, it is likely that issues not identified in this article will emerge and some of those discussed herein will evolve. Investigators and IRBs should continue to explore and discuss issues raised by citizen scientists and to develop guidelines or best practices for involving laypeople in the conduct of research on human subjects.

## Footnotes

1 Normally, institutions can handle these investigations. However, serious problems may occur when institutional officials try to cover up or ignore allegations. In such cases, those who make charges of misconduct may have obligations to the broader research community to ensure that they are addressed.

2 A test result produced by a laboratory certified according to procedures that comply with the Clinical Laboratory Improvement Amendments would be an example of a result from a well-validated test (Beskow and Burke 2010). Tests that are not well validated may produce a high percentage of false positive or false negative results.

3 Oversight would not be needed if a study qualifies as exempt from the federal regulations, for example.
